# Melatonin and Related Compounds: Antioxidant and Anti-Inflammatory Actions

**DOI:** 10.3390/antiox11030532

**Published:** 2022-03-10

**Authors:** Maria Bantounou, Josip Plascevic, Helen F. Galley

**Affiliations:** School of Medicine, Medical Sciences and Nutrition, University of Aberdeen, Aberdeen AB24 3FX, UK; m.bantounou.20@abdn.ac.uk (M.B.); j.plascevic.20@abdn.ac.uk (J.P.)

Melatonin, an indoleamine derived from tryptophan and produced in the pineal gland and other tissues [1,2] is a potent antioxidant and inflammatory agent [1]. Specifically, the aromatic indole ring of melatonin acts as a buffer and scavenges reactive oxygen (ROS) and nitrogen species (RNS). This antioxidant scavenging effect is multiplied by the downstream antioxidant activity of its reaction products and metabolites in a cascade-like manner. Furthermore, melatonin induces endogenous antioxidant enzymes, including superoxide dismutase, catalase, glutathione peroxidase, and glutathione reductase. Additionally, melatonin can dampen the activation of the transcription factor nuclear factor-kappa B (NFĸB), leading to the decreased expression of inflammatory mediators, such as cytokines, enzymes, and adhesion molecules [2]. Melatonin can be safely administered as an exogenous therapy and has the potential to be beneficial for a variety of inflammatory conditions. This Special Issue focuses on the antioxidant and anti-inflammatory actions of melatonin in both in vitro and pre-clinical studies, and reports on its translational potential as a novel therapeutic intervention in eight original articles and four reviews.

The first paper, by Gonzaléz-Candia et al., investigated the effects of the post-natal administration of melatonin in lambs subjected to gestational hypoxia as a model of pulmonary arterial hypertension of new-borns, specifically evaluating the cardiac function and right ventricle oxidative stress [3]. Neonatal lambs received either melatonin or vehicle for 21 days after birth. It was found that melatonin treatment reduced neonatal right-ventricle oxidative stress, by reducing mitochondrial and NADPH oxidase-mediated ROS production, with the upregulation of catalase and manganese superoxide dismutase. The pulmonary arterial pressure was reduced in the lambs which were administered melatonin, notably to below the cut-off value for a diagnosis of pulmonary hypertension in the clinical setting [4]. This translational study presented evidence to support the use of the post-natal melatonin treatment for arterial pulmonary hypertension of new-borns, providing a novel treatment option for babies suffering from chronic hypoxia related to cardiovascular diseases. Another recent study demonstrated that post-natal melatonin increased the activity and expression of vasodilating prostanoids in small pulmonary arteries, also supporting the use of melatonin for this condition [5].

Peroxisomal activity is important for lipid metabolism and β oxidation, which are needed for oocyte and embryo development. Both melatonin and phytanic acid have a role in this, as well as in the nuclear factor erythroid-derived 2-like 2 (referred to as Nrf2 or NFE2L2) signalling pathway. In a study by Kim et al., the interaction between peroxisomal activity and the Nrf2/NFE2L2 pathway, and the interaction with melatonin combined with phytanic acid in porcine embryos, was investigated [6]. The group used targeted siRNAs to silence the peroxisome biogenesis factor Pex19. In the absence of the siRNA, melatonin and phytanic acid treatment augmented embryonic development by increasing the peroxisomal activity, lipid metabolism, and Nrf2/NFE2L2 signalling. These effects were opposed by the Pex19-targeted siRNA. Therefore, the advantageous effects on the embryonic development of the Nrf2/NFE2L2 signalling pathway required peroxisomal activity. This activity was shown to be increased as a result of melatonin and phytanic acid, which has the potential to improve the development of porcine embryos. This has translational implications for transgenic pig production for xenotransplantation. A recent meta-analysis supported these findings, also suggesting that melatonin increases porcine embryo blastocyst rate [7].

Decreased levels of endogenous nocturnal melatonin and its primary metabolite (6-hydroxymelatonin sulphate) are associated with an increased risk of various types of cancers, including liver cancer. Treatment with melatonin promotes tumour cell apoptosis and impedes cancer cell proliferation, motility, and invasiveness, thus having the potential to improve outcomes in patients with liver cancer [8,9]. Fernández-Palanca et al. conducted a systematic review of the studies for which melatonin was used as an anti-tumour agent in liver cancer, focusing on the anti-tumour molecular and cellular mechanisms of melatonin in hepatocellular carcinomas and cholangiocarcinomas [10]. The authors report results from in vitro liver cancer cells and in vivo liver animal cancer model studies. They suggested that the anti-tumour effects of melatonin may stem from a combination of its antioxidant, chronobiotic, immune-modulating, apoptotic, angiogenesis-limiting, and autophagy-promoting properties. Furthermore, melatonin was particularly effective when used concomitantly with other antitumor therapies. The authors concluded that melatonin has potential as a treatment for liver cancer, both on its own and in combination with other anti-tumour agents. Additional research, however, is required to explore the effectiveness of melatonin against liver cancer in a clinical trial setting.

In another cell study, Guerra-Librero et al. investigated the oncostatic mechanism of melatonin in cancer cells [11]. Two different head and neck squamous cell carcinoma cell lines were treated with a range of high doses of melatonin for up to five days. It was shown that melatonin treatment resulted in changes in mitochondrial morphology and function and altered fission and fusion activities as a result of a shift towards aerobic mitochondrial metabolism. The authors concluded that melatonin adjunct treatment may have potential as a novel therapy in patients with cancer. This work was further corroborated by Guerra et al., who highlighted the oncoprotective, drug/radiotherapy-sensitising, and anti-tumour properties of melatonin in head and neck squamous cell carcinomas. These were attributed to the antioxidant and epigenetic effects of melatonin [12].

Likewise, melatonin has been shown to reduce the viability of pancreatic stellate cells (PSCs), key contributory cells in pancreatic fibrosis, inflammation, and cancer growth. Melatonin may also induce pancreatic cancer cell apoptosis by modulating cellular oxidative stress, heat shock proteins, and vascular endothelial growth factor expression [13]. Curiously, PSCs do not express membrane melatonin receptors, making the mechanism of the action of melatonin on these cells perplexing. Estaras et al. examined this mechanism using PSCs prepared in hypoxic conditions and treated with a range of melatonin concentrations [14]. It was demonstrated that melatonin inhibited inflammatory proteins and promoted ROS generation from PSCs in a concentration-dependent manner, decreasing the ratio of reduced-to-oxidized levels of glutathione (GSH/GSSG ratio). This activated protein kinase C, leading to the stimulation of the Nrf2/NFE2L2 pathway and the production of antioxidant enzymes. Therefore, melatonin may represent an anti-fibrotic therapeutic option, which could diminish the PSC-induced fibrosis, a process that contributes to pancreatic tumour growth and inflammation.

The relationship between melatonin and inflammation has been extensively investigated, especially in NOD-like receptor protein (NLRP3) inflammasome-mediated diseases. The NLRP3 inflammasome is a cytosolic multiprotein comprised of the NOD-like receptor, and adaptor and effector proteins that trigger the release of specific cytokines (e.g., IL-1β and IL-18) and initiate the innate immune system cascade, having an established role in the pathogenesis of numerous inflammatory conditions. As such, the inhibition of the NLRP3 inflammasome by melatonin as a result of its anti-inflammatory properties, could advance treatment options for a range of inflammatory conditions [15,16].

In such a context, Arioz et al. summarised the in vitro and in vivo studies that assessed this effect of melatonin on NLRP3 inflammasome-associated conditions [15]. It was concluded that melatonin can inhibit NLRP3 by regulating non-coding RNAs and has an overall anti-inflammatory effect with the inhibition of inflammatory pathways, such as NFκB, and the activation of mitophagy and the Nrf2/NFE2L2 pathway, diminishing ROS generation. The authors concluded that melatonin ought to be explored for the treatment of neurodegenerative, cardiac, pulmonary, gastrointestinal, and metabolic inflammasome-mediated diseases.

In a related study, Farré-Alins et al. examined the mechanism by which melatonin regulates the NLRP3 inflammasome, focusing on the α7 nicotinic receptor (nAChR) pathway associated with autophagy [17]. The results from in vitro studies using murine microglial cells indicated that melatonin increased the autophagic degradation of inflamed cells and reduced pro-inflammatory markers in wild-type cells. These effects were not observed in α7 nAChR knock-out mice. Similar results were achieved in vivo where in wild-type mice, melatonin reverted cognitive decline, reduced NLRP3 levels, and increased autophagic degradation. This was not observed in α7 nAChR knock-out mice. Such results imply that melatonin could play a role in the signalling between α7 nAChR and autophagy. Using melatonin to reverse autophagic degradation in microglia may clarify the biochemical pathways of this process. In turn, this may advance research a step closer to developing novel agents that can supress neuroinflammation, improving the management of neurodegenerative conditions. These outcomes were supported in a study concerning neonatal rats, where the effects of melatonin on white matter damage were investigated [18]. It was shown that, in addition to enhancing mitochondrial autophagy, melatonin treatment also inhibited Toll-like receptor (TLR)-4 and NFκB pathway signalling, thus limiting NLRP3 inflammasome activity.

Melatonin has previously shown to have anti-ageing properties with the potential to reduce skeletal muscle frailty and extend physical performance [19]. These effects of melatonin were evaluated in NLRP3 knock-out mice in a study by Sayed et al. The group aimed to explore the effects of melatonin on microRNA (miRNA) expression and inflammation in the skeletal muscle of ageing mice [20]. Melatonin was administered orally in the mouse diet and the expression of inflammatory-related miRs in the gastrocnemius muscles of wild-type and NLRP3-knockout mice were analysed. This study was the first to report the increased expression of inflammatory miRs, which was associated with increased pro-apoptotic markers in ageing wild-type mice and was reduced as a result of melatonin administration. Additionally, the benefits of melatonin were observed in skeletal muscle histology with reduced collagenous tissue and preserved muscular architecture in melatonin-treated mice. The results from this translational study support the hypothesis that melatonin may reduce inflammation and ageing-induced skeletal muscle changes.

Melatonin has also been explored as a treatment option for another inflammatory condition, infectious orchitis, which is the result of testicular bacterial infection that can cause inflammation and temporary or permanent infertility. It has been shown that melatonin reduced testicular damage in spermatogonial stem cells, by preventing pathological reactions associated with endoplasmic reticulum stress and inflammation [21]. Deng et al. further investigated the anti-inflammatory potential of melatonin both in vivo and ex vivo in orchitis [22]. A lipopolysaccharide (LPS)-induced infectious orchitis sheep model and isolated testicular tissue were used. The outcomes from the in vivo experiments confirmed that LPS reduced the testosterone content in sheep testis, and that melatonin augmented the phagocytic activity of testicular macrophages whilst reducing the generation of pro-inflammatory cytokines. Further ex vivo studies confirmed this whilst also suggesting that melatonin exerts its effects via repressing NFκB through inhibition of the p38MAPK pathway and upregulation of antioxidants. Overall, melatonin reduced testicular inflammation and enhanced testosterone production from Leydig cells. Therefore, melatonin could reduce inflammation and preserve testosterone production in infectious orchitis, justifying an investigation of the effect of melatonin in other genitourinary conditions that reduce testosterone production, such as sexually transmitted diseases and infections of the non-reproductive systems.

During the current pandemic, melatonin has been widely reported as being a potential therapeutic option against severe acute respiratory syndrome coronavirus 2 (SARS-CoV-2) referred to as COVID-19 [23,24,25]. The rationale for using melatonin against this condition stems from the elevated oxidative activity and production of ROS and RNS observed in patients with COVID-19 [26,27]. In a narrative review, Ramos et al. described the rationale for the use of melatonin, including the pathophysiology of coronavirus infection, the antioxidant and anti-inflammatory actions of melatonin, and the specifics of treatment potential in patients with COVID-19 [28]. A role for melatonin in sleep disturbance, delirium, inflammation, and mitochondrial dysfunction in patients with COVID-19 is presented. Usefully the paper also highlights differing regulatory requirements for the use of melatonin. Several trials of melatonin as an adjuvant treatment for COVID-19 have been published, although the quality of some these studies is not ideal and further larger high quality trials are needed [29,30,31,32].

Wongchitrat et al. also reviewed the role of melatonin in COVID-19, focusing on the neuroprotective effects of melatonin on COVID-19-induced neurological symptoms [33]. The protective role of melatonin that stems from its antioxidant, anti-apoptotic, and anti-inflammatory effects against a range of neurotropic viral infections was summarised. The types of viral infections resulting in neurological symptoms or neuropathogenesis, including coronavirus, were described, along with the actions of melatonin both in vitro and in vivo, and the potential mechanisms. This review and further published work [34,35,36] suggests the anti-viral activity of melatonin, supporting its use in patients with COVID-19.

In addition to the recognised physiological role in mammals, melatonin also has a role in plant growth and development. However, its specific role in chloroplast function is not thoroughly understood. Chloroplasts are vital organelles for plant survival, producing energy through photosynthesis, contributing to growth and modulating responses to environmental stresses, such as heat, chilling, salinity, drought, and pathogen invasions [37]. Lee and Back conducted a study exploring the role of melatonin in the regulation of chloroplast protein quality control (CQPC), which is responsible for the production of starch, a non-structural carbohydrate that stores energy in plants, an essential component of plant metabolism and growth [38,39].

In the study, two *Arabidopsis thaliana* cell lines were compared [38]. The first was a mutant missing the serotonin N-acetyltransferase enzyme, crucial for melatonin biosynthesis in plants, while the second was wild-type Arabidopsis. The mutant line was of a smaller size, had delayed flowering, and a lower starch composition compared to the wild type. Expression of proteins needed forphotosynthesis CQPC and ROS defence were also reduced. Notably, the introduction of melatonin stimulated the production of the photosynthesis-related proteins in the mutant line. Therefore, these results suggest that melatonin may upregulate CQPC and, by extension, starch synthesis. Diminished levels of melatonin led to plant dwarfism, delayed flowering, and low starch levels. This is of particular importance for both the food and non-food sector, as starch is the primary carbohydrate source in humans and has applications as a renewable raw material in industry [39].

In this Editorial, we present the studies that contributed to this Special Issue on the antioxidant and anti-inflammatory actions of melatonin. The breadth of the studies highlights the range of the potential beneficial effects of melatonin. The jigsaw of the complex functional mechanisms by which melatonin exerts its effects is slowly revealing the complete picture.
